# Deep Vein Thrombosis Prophylaxis in Trauma Patients

**DOI:** 10.1155/2011/505373

**Published:** 2011-05-15

**Authors:** Serdar Toker, David J. Hak, Steven J. Morgan

**Affiliations:** ^1^Orthopaedic Trauma Research, Denver Health, University of Colorado, 777 Bannock Street MC 0188, Denver, CO 80204, USA; ^2^Denver Health, University of Colorado, 777 Bannock Street MC 0188, Denver, CO 80204, USA

## Abstract

Deep vein thrombosis (DVT) and pulmonary embolism (PE) are known collectively as venous thromboembolism (VTE). Venous thromboembolic events are common and potentially life-threatening complications following trauma with an incidence of 5 to 63%. DVT prophylaxis is essential in the management of trauma patients. Currently, the optimal VTE prophylaxis strategy for trauma patients is unknown. Traditionally, pelvic and lower extremity fractures, head injury, and prolonged immobilization have been considered risk factors for VTE; however it is unclear which combination of risk factors defines a high-risk group. Modalities available for trauma patient thromboprophylaxis are classified into pharmacologic anticoagulation, mechanical prophylaxis, and inferior vena cava (IVC) filters. The available pharmacologic agents include low-dose heparin (LDH), low molecular weight heparin (LMWH), and factor Xa inhibitors. Mechanical prophylaxis methods include graduated compression stockings (GCSs), pneumatic compression devices (PCDs), and A-V foot pumps. IVCs are traditionally used in high risk patients in whom pharmacological prophylaxis is contraindicated. Both EAST and ACCP guidelines recommend primary use of LMWHs in trauma patients; however there are still controversies regarding the definitive VTE prophylaxis in trauma patients. Large randomized prospective clinical studies would be required to provide level I evidence to define the optimal VTE prophylaxis in trauma patients.

## 1. Introduction

Deep vein thrombosis (DVT) and pulmonary embolism (PE), known collectively as venous thromboembolism (VTE), affect an estimated 900,000 people in the U.S. each year resulting in several hundred thousand hospitalizations and about 300,000 deaths [1]. About two-thirds of episodes manifest as DVT and one-third as PE with or without DVT [2, 3]. In general surgical patients without prophylaxis against VTE, the incidence of DVT has been reported to be as high as 30%, with an associated fatality risk of 1% [4].

Venous thromboembolic events are also common and potentially life-threatening complications after traumatic injury [5–10]. Coagulopathy is present immediately at admission in 25% of trauma patients, and it is associated with a 5-fold increase in mortality [11]. Sevitt and Gallagher's [12] autopsy study of 125 patients revealed a 65% incidence of DVT and 16% incidence of PE. The incidence of DVT varies from 5 to 63% in trauma patients depending on patient's risk factors, modality of prophylaxis, and methods of detection [13, 14]. A general population study that followed 21,680 persons for occurrence of venous thrombosis over 7.6 years demonstrated that trauma was only present in 6%, revealing a relatively low potential number of cases globally that could be avoided with prophylaxis in this setting, while cancer was present in 48% and surgery was present in 42% [14]. Shackford et al. [15] also reported of an incidence of 7% in the high-risk trauma patient. Nevertheless two factors suggest an increasing incidence of thromboembolism after trauma. First, the average age of the population increases [16]; second, improvements in several fields have resulted in the survival of more seriously injured patients who are at high risk for VTE [16, 17]. Therefore VTE prophylaxis is warranted for patients sustaining traumatic injuries [9, 13, 18–20]. Without thromboprophylaxis, the rate of venous thrombosis and subsequent PE is substantial [18]. 

The optimal VTE prophylaxis strategy for trauma patients with a contraindication to pharmacological prophylaxis because of a risk of bleeding is unknown [6–9]. Methods of prophylaxis and detection continue to improve; however, a significant proportion of patients still develop VTE [8, 21]. In addition, the difficulty in determining optimal treatment is further complicated by the presence of occult DVTs at a 60% rate of occurrence [17, 22]. In this paper we aim to investigate the various approaches for VTE prophylaxis in trauma patients and report what appears to be the best practice for optimal VTE prophylaxis in trauma patients. A detailed literature search was completed to extrapolate articles that described DVT and DVT prophylaxis. Specific search terms used included DVT, risk factors, trauma, guidelines, and prophylaxis.

## 2. Risk Factors for DVT in Trauma Patients

Numerous different factors have been cited in the literature as posing a high risk for VTE in trauma patients. Several prospective studies have examined the risk of DVT after trauma [9]. Traditionally, pelvic and lower extremity fractures, head injury, and prolonged immobilization have been considered risk factors for VTE [8]. 

Tissue factor, which is abundant in the brain, plays an important role in initiating coagulopathy following head trauma. Early coagulopathy after traumatic brain injury has been thought to be the result of the injury-mediated local release of TF which activates the extrinsic pathway of blood coagulation [23]. However, Geerts et al. [9] in their prospective clinical trial found that major head injury was not associated with DVT. They also did not find significant association between DVT and sex, injury severity score (ISS), pelvic fracture, and amount of blood transfused. They reported that the independent predictors of DVT were age, blood transfusion, surgery, fracture of the femur or tibia, and spinal cord injury. According to this study the presence of blood transfusion was important, but the amount of transfusion was not associated with DVT. Kudsk et al. [17] also did not find an increase of DVT incidence with injury severity score but they found that it increased with increasing age. 

The effect of blood transfusion alone on DVT development has also been investigated. In a retrospective cohort study, Spinella et al. [24] found that in trauma patients transfused 5 or more units of RBCs, DVT and in-hospital mortality was increased with the transfusion of old RBCs when compared with a group of patients similar severity of injury who were transfused RBCs of decreased storage age. 

Knudson et al. [3] in an analysis of 450,375 patients in the American College of Surgeons (ACS) National Data Bank found six factors to be independently significant for VTE in trauma patients. These factors were age ≥ 40, lower extremity fracture with AIS (abbreviated injury score) ≥ 3, ventilator days > 3, head injury with AIS ≥ 3, venous injury, and a major operative procedure. 

Patients with acute spinal cord injury (SCI)) and paralysis are at considerable risk for developing DVT [25–27]. Fujii et al. [25] using radiofibrinogen uptake test in a group of patients with SCI found the overall incidence of DVT to be as higher as 57%.

In general, older age increases risk; however, the specific age at which risk increases is unclear [8]. Selby et al. [10] in their prospective cohort study found that the increasing age was the single most important independent predictor of VTE in trauma patients. The reasons for an increased thrombosis risk with age are not understood, but may relate to increasing presence of other illnesses predisposing to thrombosis, to increase in coagulation potential, or some combination of these [2].

Despite these numerous risk factors associated with the development of DVT, it is unclear which individual or combination of risk factors defines a high-risk group [22]. A risk assessment profile (RAP) score was developed by Greenfield et al. [28] (Table 1). The study by Gearhart et al. [22] supported the results of this pilot study which showed patients with a RAP score of 5 or more were 3 times more likely to experience the development of VTE than patients with a RAP score of less than 5.

## 3. DVT Mechanism in Trauma

For thrombus formation, three important factors, blood flow, blood component and blood vessels, have been recognized as Virchow's triad [29]. Major trauma often precipitates one or all of these risk factors in Virchow's triad of hypercoagulability, endothelial injury, and venous stasis [20]. According to Hak [16], trauma patients often have all three of these factors leading to a high risk of thromboembolism. Direct injury to blood vessels can cause intimal damage leading to thrombosis and prolonged bed rest, immobilization, hypoperfusion, and paralysis, all promote venous stasis [8]. Decreased levels of antithrombin III [30, 31] and suppression of fibrinolysis [32, 33] may lead the trauma patient to become hypercoagulable. In a recent study, Okamura et al. [34] found that plasma levels of D-dimer and soluble fibrin monomer complex was higher than normal in hip fracture patients. Peetz et al. [35] also showed more increased levels of D-dimer in high-risk orthopaedic surgery patients when compared with low-risk group.

Several studies have established that thromboplastin (Tissue factor; TF) and markers of thrombin generation increase after trauma [36–38] and that levels of natural anticoagulants such as antithrombin (AT), protein C (PC) and protein S (PS) are reduced [31, 37]. Selby et al. [10] concluded that major trauma leads to significantly increased and persistent thrombin with disruption of its regulation. Increased severity of hypoperfusion was associated with an increase in plasma thrombomodulin and a reduction in protein C levels. This suggests that acute coagulopathy is due to systemic anticoagulation through activation of the protein C pathway [39]. 

Besides consumption of clotting factors, acidosis and hypothermia leading to reduced activity [39], and dilution from intravenous fluids and packed cell administration [39, 40] are also accepted causes of traumatic coagulopathy. However regarding the early phase of coagulopathy in trauma, Brohi et al. [39] stated that acute traumatic coagulopathy is not due to coagulation factor consumption or dysfunction because of acidosis, moderate hypothermia, or dilution. They stated that shock itself is associated with a coagulopathy that is due to the systemic activation of anticoagulant and fibrinolytic pathways.

Immobility is also a recognized cause of VTE. Immobility due to paralysis is one of the major contributing factors for the development of DVT in patients with trauma to the spinal cord [25]. The lack of pumping action of the contracting muscles results in blood flow reduction and pooling of blood in the intramuscular sinuses of the calf, leading to DVT [41]. Increased hematocrits, elevated fibrinogen, and von Willebrand factor macromolecular complex levels increase blood viscosity [42, 43], and this may further influence blood flow [44]. Decreased blood flow could lead to endothelial damage, local accumulation of activation products of coagulation, and local decrease in inhibitor levels, all increasing coagulability of the blood [25]. According to Meissner et al. [36], associations with immobilization and obesity suggest that VTE after injury is a systemic hypercoagulable disorder with local manifestations of thrombosis related to lower extremity stasis.

## 4. Prophylaxis for DVT in Trauma Patients

Most methods of VTE prophylaxis that are effective in nontrauma patients are ineffective for multiply injured patients [8, 16] because the factors leading to thrombosis likely develop immediately after the injury, before administration of any type of prophylactic therapy is possible [16, 20]. Additionally, contraindications arising from associated injuries often limit the potential options for prophylaxis in patients with trauma [16]. However it is reported that without thromboprophylaxis overall DVT rates exceed 50% [9, 17, 45]. 

**Figure 1:**
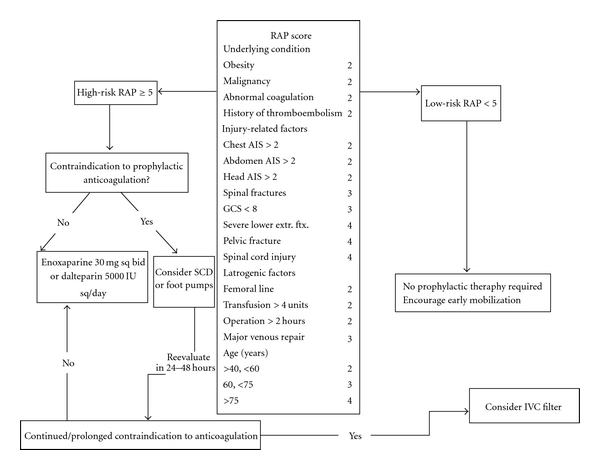
Algorithm for VTE prophylaxis [8].

Multiple studies have demonstrated the efficacy of DVT prophylaxis [3, 9, 22, 46, 47], and treatment algorithms have been established (Figure 1). However definitive randomized controlled clinical studies of prophylactic measures in trauma patients with multiple injuries are limited [3] since this heterogenous population necessitates a very large study size, which rarely has been achieved in previous studies [16]. Thus controversy exists as to the optimal method of prophylaxis in patients following trauma [16, 46, 47]. Modalities available for trauma patient thromboprophylaxis are classified into pharmacologic anticoagulation, mechanical prophylaxis, and inferior vena cava (IVC) filters [18, 48].

## 5. Pharmacologic Prophylaxis

### 5.1. Low-Dose Heparin (LDH)

A review of the general surgical literature shows that the incidence of DVT can be diminished by as much as 20% to 40% with minidose prophylactic heparin [49]. Low-dose heparin (LDH) given in doses of 5,000 units subcutaneously two or three times daily, represents one pharmacologic treatment modality for prophylaxis against DVT/PE [50]. Ruiz et al. [20] pointing out that these data have since been applied to trauma patients without substantiation [17], studied on 100 consecutive patients with multiple trauma in order to determine the efficacy of low-dose heparin and found that it did not provide adequate protection in trauma patients with an ISS > 10. Ganzer et al. [51] also concluded that for the prophylaxis of thromboembolic complications especially in the high-risk areas of orthopedics and trauma surgery, unfractioned standard heparin (UFH) is insufficiently effective and associated with a high risk of side-effects. Geerts et al. [52] in a randomized, double blind, prospective trial comparing LDH with LMWH found that LDH was significantly insufficient in DVT prevention. In some other studies comparing LDH with no prophylaxis [3, 15, 53–56], no significant difference was reported. Despite the similar results showing the inefficacy of LDH in all these studies, a recent study by Arnold et al. [57] showed comparable rates of DVT in trauma patients receiving standard-dose 30 mg bid enoxaparin versus 5000 U *three times a day* heparin. The authors concluded that in trauma patients, subcutaneous heparin dosed three times a day may be as effective as standard-dose 30 mg bid enoxaparin for VTE prophylaxis without increased complications, and this was associated with significant pharmaceutical cost savings.

### 5.2. Low Molecular Weight Heparin (LMWH)

LMWHs are generated from the chemical depolymerization of unfractioned heparin (UH). This reduces their size, charge, and weight. Secondary to their smaller size, LMWHs have significantly greater activity towards factor Xa than UHs [58]. 

LMWHs gained popularity as prophylactic agents for VTE in early 1990s [59] and have emerged in the late 1990s as the most (or only) effective method of DVT prophylaxis in trauma patients [8, 50]. Multiple studies have investigated the optimal method of prophylaxis for DVT, recognizing that DVT and PE rates are lowered in trauma patients who are treated with LMWH [46, 52, 60–63]. In 1990, Green et al. [62], in a study of patients with spinal cord injury, found that LMWH was safe and effective for VTE prevention in complete motor paralysis and was superior to subcutaneous heparin. In one of the most cited studies on this topic, Geerts et al. [52] comparing LDH with LMWH found an incidence of 44% and 31% (*P* = .014) of DVT, and 15% and 6% (*P* = .012) of proximal DVT respectively. They concluded that LMWH should be considered the method of choice for the prophylaxis of trauma patients. In 1994 Knudson et al. [60] showed that LMWH (enoxaparin 30 mg bid) was safe and effective in preventing DVT in high-risk trauma patients. In 1998, American College of Chest Physcians (ACCP) recommended DVT prophylaxis in multiple trauma patients with LMWH for the first time. In 2007, Cothren et al. [46] in a prospective study including 6247 trauma patients found that once-daily dosing of prophylactic LMWH dalteparin was feasible, safe, and effective in high-risk trauma patients allowing to “operate through” systemic prophylaxis and ensuring timely prophylaxis for brain-injured and multisystem trauma patients. In a recent study, Sems et al. [64] favored the protocol of early joint spanning external fixation with the concurrent use of LMWH in patients with high-energy lower extremity trauma. They found a 2.1% incidence of DVT on duplex ultrasound examination and concluded that this incidence does not exceed historical controls.

There is a controversy in dosing of LMWHs. Some authors [35, 59] suggested dose adjustment according to the levels of D-Dimer in prevention of DVT in trauma patients. [65] found no difference in the incidence of DVT between the patients who received standard and body-weight-adjusted dose of LMWH. However a recent study by Malinoski et al. [66] showed that standard dosing of enoxaparin leads to low anti-Xa levels which are associated with a significant increase in the risk of DVT in half of surgical intensive care unit (ICU) patients, and authors concluded that these data support future studies using adjusted-dose enoxaparine.

In severely injured patients, VTE remains a major cause of potentially preventable death despite considerable prophylactic efforts particularly in those who are believed to have contraindications to receiving a heparin drug [8, 21]. Because of the fact that safer and more effective prophylactic measures are needed for high-risk trauma patients, search for new solutions is ongoing.

### 5.3. Fondaparinux

 Fondaparinux is a nonheparin drug and the first synthetic pentasaccharide selectively inhibiting Factor Xa [21, 67, 68]. Along with its antithrombotic efficacy in preventing VTE after elective orthopaedic operations and in selected high-risk abdominal surgical patients [21], the safety of fondaparinux has been documented in several Phase II and III clinical trials [68]. Two studies were performed to detect the efficacy of fondaparinux in hip fracture surgery [69, 70]. In the first study by Eriksson et al. [69] in 2001, a risk reduction of 56.4% was found in the fondaparinux group versus enoxaparine group. The second study by the same group in 2004 revealed a 1.4% incidence of DVT following a hip fracture surgery [70]. Despite these studies, Fondaparinux has never been used in trauma patients until a pilot study was performed by Lu et al. [21] in 2009. In this study, authors found a 4.6% overall incidence of DVT and a 1.2% incidence of DVT in patients receiving fondaparinux. There were no episodes of pulmonary embolism, thrombocytopenia, or bleeding attributable to fondaparinux. Authors concluded that this agent appears to offer protection against VTE in high-risk trauma patients. Its once-daily dosing regimen can improve compliance and reduce cost and eliminate risk of heparin-induced thrombocytopenia. As this is a pilot study with a lack of a control group and with a relatively small size, further research on this agent is needed.

## 6. Mechanical Prophylaxis

Various types of external compression devices are available to provide DVT prophylaxis in the immobilized patient. These devices include graduated compression stockings (GCS), sequential pneumatic compression devices (PCDs), and pneumatic plantar (A-V) foot pumps [16]. They function by reducing the luminar diameter of a vein resulting in an increase in venous flow velocity, and they are commonly utilized in trauma setting because of ease of use and inherently low risk of associated bleeding [18].

### 6.1. Graduated Compression Stockings

GCSs are widely used for prevention and treatment of DVT in nontrauma patients [71–77]. In a recent systemic review of 18 randomised clinical trials (RCTs), Sachdeva et al. [78] concluded that GCSs are effective in diminishing the risk of DVT in hospitalized patients; however, these devices have not been reported in the trauma population [18].

### 6.2. Pneumatic Compression Devices

PCDs for prophylaxis against DVT has been studied and increasingly used in trauma patients [53, 56, 79–81]. Despite the findings that PCDs were comparable to the effect of LDH in significantly lowering DVT incidence compared with controls with no prophylaxis [56, 81] and despite the given same rate of DVT for clinically injured patients prophylaxed with either Sequental Compression Device (SCD), LDH, or a combination of these [79], a meta-analysis by Velmahos et al. [56] showed that PCD offered no benefit over no prophylaxis. Kurtoglu et al. [82] also prospectively randomized 120 head/spinal traumatized patients for comparison of IPC with LMWH as a prophylaxis modality against VTE. Venous duplex color-flow Doppler sonography of the lower extremities was performed each week of hospitalization and 1 week after discharge. There was no statistically significant difference regarding a reduction in DVT, PE, or mortality between groups (*P* = .04, *P* > .05, *P* > .05, resp.), and they concluded that PCD can be used safely for prophylaxis of VTE in head/spinal trauma patients.

 Mechanical VTE prophylaxis by graduated compression stockings or intermittent pneumatic compression provides suboptimal protection, and its use is recommended only in combination with LMWH prophylaxis [83] or when anticoagulant DVT prophylaxis is contraindicated [18].

### 6.3. A-V Foot Pumps

In 1983, Gardner and Fox first described the physiologic pumping mechanism of the sole of the foot and in 1990 Laverick demonstrated that arterio-venous (A-V) foot pump increases venous blood flow in popliteal vein by 250% [50]. In a recent study by Pitto and Young [76, 77], the authors found a 2.7% incidence of DVT in a group of patients following total hip or total knee arthroplasty. A-V foot pumps were studied in several trauma patient groups, and their efficacy was compared with PCD and LMWH. Knudson et al. [84] found higher rates of DVT with foot pumps when compared with PCD and LMWH, and Anglen et al. [85] found an incidence of 4% of DVT with foot pumps and 0% with PCD. However, Spain et al. [86] found no significant difference in DVT rates between the two groups, with PCDs at 7% and A-V foot pumps at 3%. In a recent study by Stannard et al. [87], enoxaparine treatment was compared with enoxaparine plus foot pumps treatment. The prevalence of deep-vein thrombosis was 13.4% for enoxaparine group, and 8.7% for enoxaparine plus foot pumps group. There were eleven large or occlusive clots (prevalence, 11.3%) in enoxaparine group, compared with only three (prevalence, 2.9%) in enoxaparine plus foot pumps group (*P* = .025). The prevalence of pulmonary embolism was 2.1% in enoxaparine group and 0% in enoxaparine plus foot pumps group. The authors concluded early mechanical prophylaxis with foot pumps and the addition of enoxaparin on a delayed basis was a very successful strategy for prophylaxis against venous thromboembolic disease following serious musculoskeletal injury.

## 7. Vena Cava Filters (VCFs)

The effectiveness of a VCF in the prevention of pulmonary embolism in patients with proximal DVT has been well established. Traditionally, these filters have been placed in patients with acute proximal DVT or a recent PE who have either a contraindication to receive heparin, who had bleeding during heparin treatment, or who have had a PE despite anticoagulation [3]. Thus in trauma patients with contraindications to chemoprophylaxis and mechanical prophylaxis [18, 50], VCFs offer one option to reduce morbidity and mortality associated with embolism.

Unfortunately VCFs are not without risks and potentially transient nature of the hypercoagulable states in the majority of trauma patients has made this a less attractive option [88]. However with concern about the ineffectiveness of available VTE prophylaxis in injured patients, some authors have advocated the placement of VCFs in high-risk patients who have neither PE nor DVT [88–92]. This is certainly controversial [55], however, a retrospective analysis of data from the National Trauma Data Bank of the American College of Surgeons by Shackford et al. [93] demonstrated that 6282 of 617,349 patients received a VCF (1%) between 1991 and 2002, and 86% of these were placed prophylactically. 

Several reports exist in the literature on the use of prophylactic VCFs in trauma patients with some of them demonstrating a significant reduction in the incidence of PE. Khansarinia [94] found significant differences in both PE and PE-related death when compared high-risk trauma patients with prophylactic Greenfield filter placement with injury-matched controls without filters. Velmahos and collegues [26] in their meta-analysis found that patients with prophylactic vena cava filters had a lower incidence of PE (0.2%) compared with those without filters (1.5%) versus historical controls (5.8%). In a recent study by Toro et al. [40], authors concluded that VCFs were safe and effective in preventing PE, and the risk of recurrent DVT was low; however, some authors reported that permanent VCFs had some disadvantages such as increasing the long-term risk of DVT [95–97]. Phelan et al. [98] in a long-term study regarding the follow-up of permanent VCF in trauma patients, concluded that permanent filters should be the choice for elderly patients. Since most of the trauma patients are relatively younger, new solutions might be required. With the approval of retrievable systems, there has been renewed interest in VCFs. The use of retrievable VCFs offers a dual advantage: first protection against PE during the risk period, and second the option of filter removal thus avoiding late complications [96]. 

Some recent studies reported good results of retrievable VCFs. Gorman et al. [97] using these filters in 113 trauma patients concluded that retrievable filters were safe and effective in preventing PE in high-risk patients. Rosenthal et al. [99] concluded that retrievable VCFs could even be placed in bedside in ICU in critically ill trauma patients and it was a safe and simple technique that was avoiding the transportation of the patient out of ICU. Cherry et al. [100] using the prophylactic retrievable VCFs in trauma patients, reported 1.6% PE rate, high retrieval rate (59%), low complication rate (0.1%), and satisfactory compliance with traditional Eastern Association for the Surgery of Trauma (EAST) guidelines.

## 8. What Do the Latest ACCP and EAST Guidelines Recommend for DVT Prophylaxis in Trauma

For more than 20 years, American College of Chest Physicians (ACCP) has published guidelines for the prevention of VTE [101, 102]. According to the latest ACCP guidelines published in 2008 [101], ACCP recommends the use of LMWH for major trauma patients as soon as it is considered safe to do so. An acceptable alternative is the combination of LMWH and the optimal use of a mechanical method. If there is a contraindication for LMWHs, mechanical thromboprophylaxis with PCD or possibly with GCS alone was recommended. For major trauma patients with impaired mobility, ACCP recommends thromboprophylaxis until hospital discharge. ACCP recommends against the use of a VCF as thromboprophylaxis for trauma and SCI patients. For patients with acute SCI, ACCP recommends thromboprophylaxis with LMWH, alternatively, combined PCD and either LDH or LMWH. If anticoagulant therapy is contraindicated, the optimal use of PCD and/or GCS is recommended.

The Eastern Association for the Surgery of Trauma (EAST) has taken a leadership role in the development of evidenced-based guidelines for trauma [103]. a practice management guidelines for the prevention of venous-thromboembolism in trauma patients were published in July 2002 (Table 2) [50]. 

### 8.1. Recommendations of EAST about LDH

EAST group has no Level I recommendation for LDH. As level II recommendation, little evidence exist to support the benefit of LDH in the trauma patient. Level III recommendation is that for patients in whom bleeding could worsen injuries, the safety of LDH has not been established, and an individual decision should be made when considering prophylaxis [50].

### 8.2. Recommendations of EAST about LMWH

EAST group has no Level I recommendation for LMWH. According to Level II recommendations LMWH can be used with the following injury patterns:

1. pelvic fractures (operative or prolonged bed rest),
2. complex lower extremity fractures (operative or prolonged bed rest),
3. spinal cord injury.

According to Level III recommendation, patients with an ISS > 9 should receive LMWH primarily.

### 8.3. Recommendations of EAST about A-V Foot Pumps

There is no Level I and Level II recommendation about A-V foot pumps because of insufficient data. According to Level III recommendation, these devices may be used as a substitute for pneumatic compression devices in those high-risk trauma patients who cannot wear PCDs.

### 8.4. Recommendations of EAST about PCDs

There is no Level I and Level II recommendation about PCDs because of insufficient data. According to Level III recommendation, in the subset of head-injured patients, PCDs may have some benefit in isolated studies

### 8.5. Recommendations of EAST about VCFs

There is no Level I and Level II recommendation about PCDs because of insufficient data. According to Level III recommendation, insertion of a prophylactic VCF should be considered in very-high-risk trauma patients who cannot receive anticoagulation because of increased bleeding risk and have to be immobilized for a long time [50].

## 9. Conclusions

It is clear that VTE is one of the major problems of trauma patients. As demonstrated in the extended body of literature on prophylaxis of VTE in trauma patients, there is an insufficiency of high-qualified clinical studies to let clinicians to decide the definite way for prophylaxis in this group of patients. Despite the fact that none of the methods of the prophylaxis provide complete prevention from VTE, it is clear that without prophylaxis the incidence of occult and nonoccult DVT would be higher with the potential for increased risk of VTE-related morbidity and mortality.

Most recent clinical studies advocate the use of LMWHs in the prevention of VTE in trauma patients; two important guidelines, ACCP and EAST, also recommend primary use of LMWHs in trauma patients. However, according to new research we think that LDH may gain importance again as it is shown that it might be as effective as LMWHs in trauma patients with an adjustment of dosage and that might provide an advantage of lower costs. Larger and qualified studies are required to be able to recommend the usage of LDH again instead of LMWH. Mechanical prophylaxis was also advocated by many studies and ACCP and EAST guidelines; however it is mostly stated that this methods should mostly be used as an adjuvant therapy to LMWHs or they are recommended to be used where there is a contraindication for LMWHs. Additionally the nature of the major trauma itself can be a contraindication to use such devices such as requirement for external fixators on lower extremities would prevent application of GCSs or PCDs.

There is increasing number of studies about VCFs. Traditionally it is being used for PE prophylaxis where there is a contraindication for LMWH prophylaxis; however it is widely discussed that this device could be used for primary prophylaxis in major trauma patients. Currently EAST guidelines recommend the use of VCFs in very-high-risk major trauma patients in case of a contraindication for LMWHs. However, ACCP does not recommend the use of VCFs in major trauma patients.

In conclusion, VTE prophylaxis in trauma patients is necessary and with benefit to the patient. The diminishment of VTE with prophylaxis has not, however, completely eliminated mortality or morbidity of VTE. Large qualified randomized prospective clinical trials would be required to diminish controversy and to further determine the ideal prophylaxis for VTE in trauma patients.

##  Disclosure

The authors have nothing to disclose.

## Figures and Tables

**Table 1:** Individual risk factors and points allotted to calculate the RAP score.

Underlying condition	Points
Obesity	2
Malignancy	2
Abnormal coagulation	2
History of thromboembolism	3
Iatrogenic factors	
Femoral venous line	2
Transfusion > 4 units	2
Operation > 2 hours	2
Major venous repair	3
Injury-related factors	
Chest AIS > 2	2
Abdomen AIS > 2	2
Head AIS > 2	2
Spinal fractures	3
Glascow coma score < 8	3
Severe lower extremity fracture	4
Pelvic fracture	4
Spinal cord injury	4
Age (years)	
≥40, <60	2
≥60, <75	3
≥75	4

**Table 2:** Recommendations of EAST group for the VTE prophylaxis in trauma patients.

Prophylaxis	Level I recom.	Level II recom.	Level III recom.
LDH	None	Little evidence exist to support the benefit of LDH in the trauma patient	Individual decision should be made when considering prophylaxis
LMWH	None	(1) Pelvic fractures (2) Complex lower extremity fractures (3) Spinal cord injury.	Patients with an ISS > 9 should receive LMWH primarily
A-V foot pump	None	None	Substitude for PCDs in those high-risk trauma patients who cannot wear PCDs
PCDs	None	None	In head-injured patients, PCDs may have some benefit in isolated studies
VCFs	None	None	Very-high-risk trauma patients who cannot receive anticoagulation
